# Palliative semi-permanent abdominal drain for the management of refractory malignant ascites: a retrospective study in a comprehensive cancer center

**DOI:** 10.1007/s00520-025-09551-1

**Published:** 2025-05-24

**Authors:** Caroline Poisson, Anda Sampetrean, Perrine Renard, Rita-Maria Khoury-Abboud, Florian Scotté, Laurence Vigouret-Viant, Baptiste Bonnet, Lambros Tselikas, Frédéric Deschamps, Christine Mateus

**Affiliations:** 1 Palliative Care Unit, Interdisciplinary Department for the Organization of Patient Course (Supportive Care Department), Gustave Roussy, Paris-Saclay University, 114, Rue Edouard-Vaillant, 94800 Villejuif, France; 2 Interventional Radiology Unit, Anesthesiology, Surgery and Interventional Department, 94805 Villejuif, France

**Keywords:** Refractory malignant ascites, Palliative care, Semi-permanent abdominal drain, End-of-life, Quality of life

## Abstract

**Purpose:**

Refractory malignant ascites in the advanced palliative phase significantly impacts patients’ quality of life (QoL), causing pain, respiratory difficulties, digestive issues, and impaired mobility. While iterative drainages can effectively relieve symptoms, frequent hospital visits and the significant volume of fluid requiring removal pose considerable challenges. A semi-continuous approach using a permanent bedside drain may offer more frequent drainages of smaller volumes. This study aimed to examine the feasibility, reliability, and safety of a semi-permanent bedside abdominal drain for patients in advanced palliative care with refractory malignant ascites.

**Methods:**

This is a retrospective study, with cases identified through computerized queries of digital patient records. Data collected included patient characteristics, biological parameters, procedure details, and end-of-life outcomes.

**Results:**

Between 2019 and 2024, this drain was proposed to 25 palliative care patients. They had received a median of three lines of oncological treatment, with 60% of them receiving exclusively palliative care at the time of drainage. Drain placement had a beneficial impact on disabling symptoms in over 92% of cases, allowing 60% of patients to return to home hospitalization, without requiring additional hospital visits for paracentesis. The median time between drain placement and end of life was 36.5 days [4;147], while the median time from the diagnosis of refractory ascites to death was 93.7 days [14;263].

**Conclusion:**

A non-tunneled semi-permanent catheter, easily implanted at the patient’s bedside, may improve QoL. This study serves as a pilot for a prospective cohort that will analyze QoL improvements and economic costs.

## Introduction

The emergence of refractory malignant ascites in the advanced palliative phase significantly impacts patient’s quality of life (QoL), causing pain, respiratory difficulties, digestive issues, and impaired mobility. Malignant ascites accounts for approximately 10% of all cases of ascites and is a marker of poor prognosis [1]. Survival in this patient population is limited, with an average of approximately 20 weeks from diagnosis [2, 3]. The pathophysiology of malignant ascites is multifactorial, involving defective resorption by lymphatic capillaries due to tumor invasion, and hypersecretion of vascular endothelial growth factor (VEGF), leading to increased capillary filtration into the peritoneum. Additionally, ascites may result from portal hypertension caused by hepatic tumor infiltration [2].

Treatment of refractory malignant ascites is palliative, aiming primarily to relieve discomfort by reducing fluid volume. Several management options are available without standardized recommendations. Paracentesis remains the most common method, providing rapid but temporary symptom relief [4, 5]. Indeed, fluid reaccumulates quickly, with a median recurrence time of 10.4 days, requiring repeated paracentesis [6, 7]. Furthermore, the efficacy of paracentesis declines over time due to the progressive compartmentalization of ascitic fluid. The use of diuretics in the management of malignant ascites varies among physicians and no randomized controlled trials have assessed their efficacy. Greenway et al. described a good symptomatic control of ascites with high doses of spironolactone (150–400 mg/day) in a small group of patients with sodium retention and high plasma renin activity [8]. In addition, alternative and more specific approaches are proposed, including external drainage systems using tunneled or non-tunneled catheters, which range in complexity [9–12].

In our palliative care practice, managing recurrent ascites is a frequent issue, particularly for highly frail patients with a limited prognosis. To address this, semi-continuous drainage has been implemented using a non-tunneled, semi-permanent peritoneal catheter, allowing for more frequent but smaller-volume drainages. This approach seeks to balance the benefits of symptom relief with the invasive nature of the intervention, focusing on improving QoL.

Since 2019, we have proposed non-tunneled, semi-permanent peritoneal catheter to simplify symptomatic drainage for several patients. For patients with a very limited prognosis who are unfit for invasive procedures, tunneled permanent catheter such as PleurX® system (Denver Biomedical, Inc., part of Cardinal Health, Inc.; Golden, CO) are often deemed unsuitable due to their invasive nature.

The aim of this work was to examine the feasibility, reliability, and safety of a semi-permanent bedside abdominal drain for advanced palliative patients with refractory malignant ascites.

## Patients and methods

### Patients

The procedure was offered to adult patients with advanced cancer complicated by refractory ascites and a short-term prognosis (less than 3 months). All patients had to come more than twice a month for drainage and had to feel uncomfortable with this refractory malignant ascites.

It was not recommended for patients undergoing oncological treatments that induce aplasia, due to the increased risk of infection. Furthermore, patients were required to be under the care of a home hospitalization service to ensure proper follow-up and management of the drain.

### Study design

This was a non-interventional retrospective study. We included all patients who underwent placement of a semi-permanent peritoneal drain in the advanced palliative phase between 2019 and 2024. Cases were identified through computerized queries of digital patient records. Data collected included the following:

- *Patient’s characteristics*: age, sex, general condition according to the WHO scale, and biological parameters at the time of the procedure (lactate dehydrogenase (LDH), serum albumin levels)
- *Oncological situation*: type of cancer, tumor stage, number of treatments received, and the date of the last treatment
- *Procedure details*: date of the procedure, immediate and delayed complications, and benefits related to the procedure
- *End-of-life data*: occurrence of death, the time and place of death, events that occurred in the last month of life

This study was reviewed and approved by the Scientific Commission, which did not identify any ethical concerns. It was approved by our institutional review board (IRB N°2023–258). Written informed consent requirement was waived. The investigators declare no conflicts or interests, and the research was not funded.

### Procedure

**Fig. 1:**
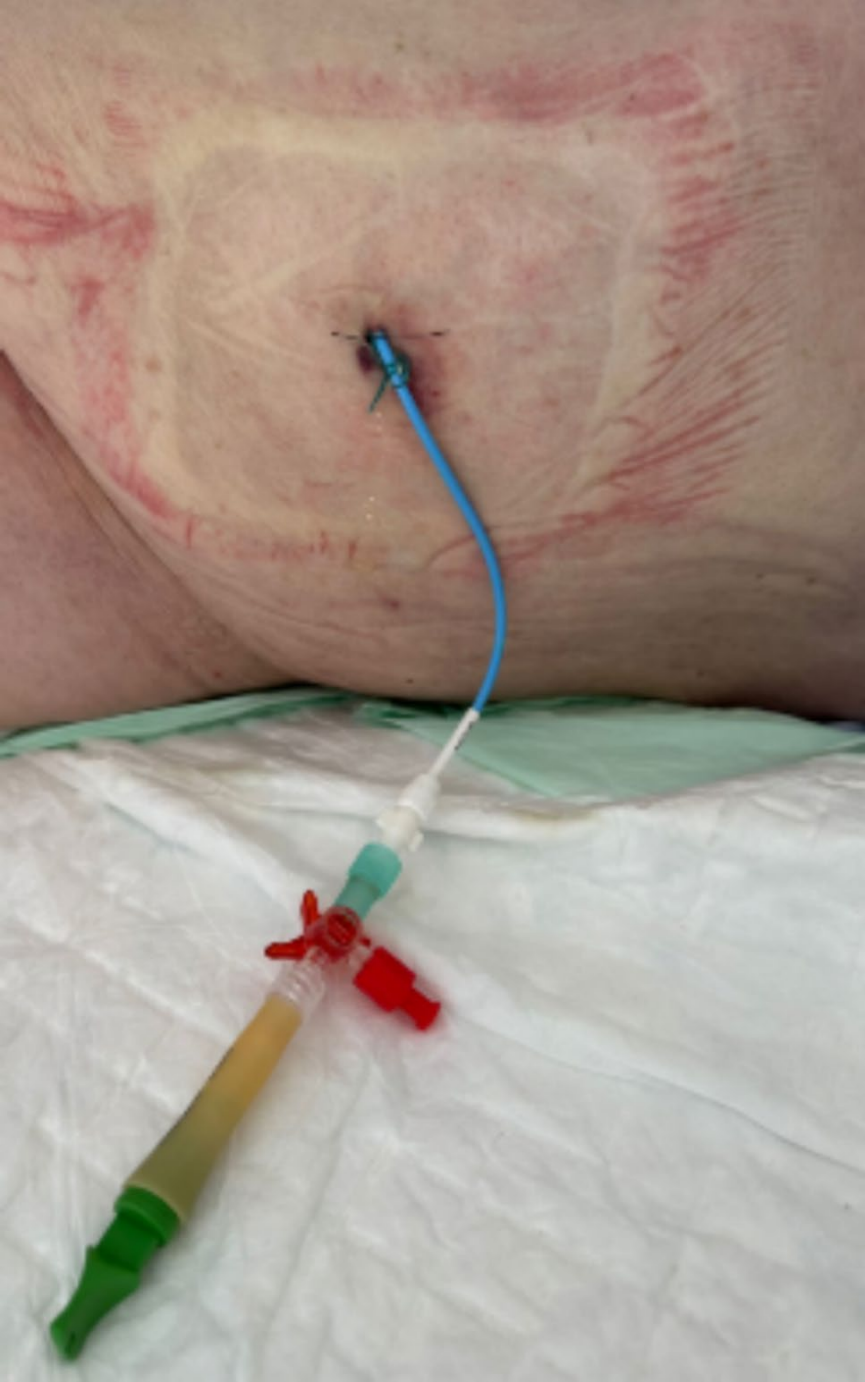
Example of a non-tunneled semi-permanent catheter

The semi-permanent drain used in our hospital is a nephrostomy drain with a diameter of 10 or 12 French drainage catheter (Flexima®, Boston Scientific, Natick, MA). The procedure is technically and logistically easy requiring no operating room. Indeed, the drain is placed with no tunneling and anchored to the skin at a single point. It is performed under local anesthesia and ultrasound guidance, either in the interventional radiology department or at the patient’s bedside (Fig. 1). Catheter fixation was achieved using a simple interrupted suture to minimize the risk of accidental catheter removal or drain dislodgment.

After the placement of the semi-permanent non-tunneled drain, drainage was performed at the patient’s bedside upon request, based on symptoms of discomfort. This procedure was carried out by a nurse either at home or during hospitalization.

### Safety

We recorded the immediate complications that occurred within the first week following placement, as well as the delayed complications (beyond one week and up until death).

## Results

The procedure was offered to 25 patients (median age 58 years) in the advanced stages of their disease. All patients had metastatic disease and had received a median of three lines of specific oncological treatment. Sixty percent of them were receiving exclusively palliative care. All patients required weekly paracentesis with high-volume drainage, ranging from 2 to 8 L each time. Frequent visits to the day hospital or regular paracentesis during hospitalization were required. The symptoms observed were consistent with those reported in the literature [13] (Table 1).

**Table 1:** Characteristics of patient. Data are expressed as *n* (%, rounded up to the next decimal place), median//mean [min;max]

Variables	No. (%)
Demographic data	
Female	15 (60)
Age, years	58//56 [26;84]
Oncological data	
Tumor type	
Gynecological	7 (28)
Breast	4 (16)
Kidney	4 (16)
Lung	3 (12)
Pancreas	3 (12)
Colorectal	2 (8)
B-cell lymphoma	1 (4)
Thymoma	1 (4)
Therapeutic goal	
Palliative chemotherapy	10 (40)
Palliative care only	15 (60)
Metastases	25 (100)
Lines of Chemotherapy	3//3.9 [0;9]
Last chemotherapy, days	20.5//27.5 [4;145]
General state	
Performance Status at inclusion	
2	10 (40)
3	11 (44)
4	4 (16)
LDH ≥ 1,5 N	6 (25)
Albumin < 33	17 (68)

The procedure was well tolerated, with no immediate severe complications. One patient had moderate leakage at the drain site, which was resolved with the application of a dressing. Another patient had the drain dislodged within 24 h post-procedure due to confusion, and it was decided not to reinsert the drain (Table 2). Three weeks after the procedure, three patients (12%) developed an ascitic fluid infection, requiring antibiotic therapy. One of these patients had an unfavorable outcome, leading to death during hospitalization due to multifactorial causes (refractory bowel obstruction, acute renal failure, and tumor progression) (Table 3). Two patients (8%) experienced disabling leakage at the drain site, necessitating the placement of a new drain, which resulted in a favorable outcome. Another patient had a dysfunctional drain 15 days after placement, but did not undergo drain reinsertion due to significant deterioration in his general condition (Tables 2 and 3).

**Table 2:** Symptoms, benefits, and complications related to placement. The data are expressed in *n* (% rounded up to the nearest decimal)

Variables	No. (%)	
Symptoms before placement*		
Pain and discomfort	21 (84)	
Respiratory discomfort	7 (28)	
Digestive symptoms (vomiting, nausea, subocclusive syndrome)	8 (32)	
Anxiety	1 (4)	
Recurrence of ascites < 1 week	25 (100)	
Immediate complications	2 (8)	
Drain fall out within 24 h	1 (4)	
Leaks at the orifice	1 (4)	
Delayed complications	7 (28)	
Infections	3 (12)	
Leaks at the orifice	1 (4)	
Dysfunction of the drain upon mobilization	3 (12)	
Benefits		
Return to home hospitalization	15 (60)	
Improvement of symptoms of discomfort *	23 (92)	
Improvement of digestive symptoms	8 (34.8)	
Improvement of pain and discomfort	15 (65.2)	
Improvement of dyspnea	5 (21.7)	
Improvement of anxiety	1 (4.3)	
Death	22 (88)	
In home hospitalization	1 (4)	
In a palliative care unit	11 (44)	
In the acute palliative care unit of GR** [14, 15]	6 (24)	
In an oncology care unit	4 (16)	
Time from drain placement to death (in days) Time between death and refractory ascites diagnosis (in days)	Median	Mean [min;max]
	21 75	36.5 [4;147] 93.7 [14;263]

*A patient could present multiple discomfort symptoms, so the total exceeds 100%

**The primary goal of this acute palliative care unit, in addition to managing refractory symptoms, is to facilitate communication among oncologists, palliative care specialists, patients, and their families. This collaborative approach seeks to redefine the therapeutic plan, develop a life project, and prevent unnecessary and costly obstinacy

**Table 3:** Detailed characteristics of post-procedure infections

Patient	Infection onset	Bacterial isolates	Antibiotic sensitivity	Treatment	Outcome
Patient 1	2 months after insertion	*Pseudomonas putida*	Sensitive to cefepime	Drain removal and appropriate antibiotic therapy	Favorable evolution No reinfection before death
Patient 2	6 weeks after insertion	*Enterococcus faecalis* Coagulase-negative* Staphylococcus*	Sensitive to amoxicillin Resistant to amoxicillin, and intermediate resistance to levofloxacin	Amoxicillin + levofloxacin 500 mg twice daily for 15 days	Favorable evolution, no drain removal No reinfection before death
Patient 3	3 weeks after insertion	*Enterobacter hormaechei* (bacteremia) *Acinetobacter radioresistens* (in the ascitic fluid)	Sensitive to piperacillin tazobactam Intermediate resistance to ciprofloxacin	Antibiotics + PAC removal	Unfavorable evolution due to major oncological progression, multiorgan failure, septicemia, and death

Ten patients (40%) were still undergoing oncological treatment at the time of drain laying. However, these treatments did not cause aplasia (e.g., targeted therapies or monoclonal antibodies).

Drain placement had a beneficial impact on disabling symptoms in more than 92% of cases, allowing 60% of patients to return to home hospitalization (that provides acute and subacute medical care at home in an attempt to reduce the need for hospitalization), without the need for day hospital visits for ascitic drainage.

Eight patients (35%) showed improvement in digestive symptoms (less vomiting, resolution of a subocclusive syndrome, less abdominal distension); 15 patients (65%) reported less pain or discomfort after the drain placement; 5 patients (22%) experienced reduced dyspnea; and 1 patient (4%) reported feeling less anxious.

The average time between drain placement and death was 36.5 days [4;147], and the average time between the diagnosis of refractory ascites and death was 93.7 days [14;263] (Table 2).

## Discussion

This retrospective analysis suggests that semi-permanent drains could play a role in managing patients with refractory ascites in palliative care, particularly for those who are frail, reluctant to undergo invasive procedures, or have a very poor prognosis. The use of semi-permanent drains may also be considered in cases where the patient wishes to return home.

Regarding complications, the available literature reports various data, and no studies have compared different devices or methods, such as repeated paracentesis, which is the most common management approach. The complication rate observed in our cohort aligns with data reported in other studies [16, 17] and was managed without major difficulties, with the exception of one death that remains multifactorial in etiology with major oncological evolution. The use of antibiotic prophylaxis has been discussed with the infectious disease team. Primary antibiotic prophylaxis does not seem to be relevant, as there was no infection during or immediately after the drain placement procedure. We also discussed the relevance of secondary antibiotic prophylaxis, as recommended for cirrhotic patients with refractory ascites [18]. However, the situation is different in this context: malignant ascites is a protein-rich, inflammatory ascites, with a high risk of compartmentalization, which could reduce the efficacy of antibiotic prophylaxis. Moreover, our patients are often very fragile, with a high risk of *Clostridium difficile* infections (due to immunosuppression, repeated hospitalizations, and imbalance of intestinal flora due to previous chemotherapy), and the risk of such infections could be increased if we implemented secondary antibiotic prophylaxis [19]. This retrospective study was carried out to assess the feasibility and acceptability of these non-tunneled drains by all the specialists involved and the patients. Initially, interventional radiologists and infectious disease specialists were reluctant to adopt these drains because of the assumed infectious risk. Finally, they were reassured by the management of the complications. To minimize the risk of infection, we chose not to include patients undergoing myelosuppressive chemotherapy.

Our analysis indicates an improvement in comfort, symptom relief, and the possibility for patients to return home, which may be associated with an improvement in QoL [20, 21]. None of the patients required readmission to the day hospital or any further consultation due to disabling symptoms related to ascites, an important point for QoL, especially considering how exhausting hospital visits can be for patients receiving advanced palliative care. However, 40% of patients did not return home after the drain was placed. This is because the drain was inserted during hospitalization, and some patients were unable to return home due to a significantly deteriorated general condition or because they requested a transfer to a palliative care unit. Some passed away during the same hospitalization, just a few days after experiencing symptom relief. This represents one of the limitations of our study. The average time between the diagnosis of refractory ascites and death was 22.9 weeks [4.4–65.6]. However, the average time between drain placement and death was only 26.4 days [4–99], which limits the assessment of long-term benefits and complications of the procedure. Given these findings, it may be beneficial to offer the procedure earlier in the course of refractory ascites to provide more patients with the opportunity to return home.

However, our study has other several limitations. This is a monocentric study, with a small cohort, and we employed a retrospective medical record review, so the prevalence of symptoms before and post intervention may have been underreported and underestimated. In the absence of randomized studies comparing repeated ascitic drainage with continuous drainage via an indwelling catheter, there is no convincing evidence to suggest the superiority of one technique over the other in improving QoL [22]. Therefore, a prospective study comparing repeated paracentesis with semi-permanent drain placement, alongside a QoL questionnaire, would be valuable. This approach would provide a pragmatic assessment of the relative advantages and disadvantages of these methods in the specific context of end-of-life care.

In the past 5 years, we have only offered the procedure to 25 patients. This is partly due to the COVID health crisis, which impacted the hospital’s organization, particularly in terms of access to interventional radiology. However, it is important to note that 14 procedures have been performed since 2023. The pathway is now better established: interventional radiologists are less hesitant, and the medical oncology team is more informed and reassured by the process.

It should be noted that large-volume paracentesis (more than 5 L) can induce circulatory dysfunction, characterized by a marked increase in cardiac output, decreased blood pressure, and a sudden drop in right atrial pressure due to reduced intrathoracic pressure. This can lead to elevated plasma renin and aldosterone levels. Such adverse effects are associated with impaired renal function, rapid reaccumulation of ascites, and shorter survival [23–25]. This complication is well documented in cirrhotic patients. It seems reasonable to hypothesize that in palliative patients with refractory ascites of mixed etiology, including portal hypertension (with mechanisms similar to cirrhosis), performing iterative low-volume punctures or “semi-continuous” drainage could be better tolerated hemodynamically, with slower recurrence of ascites. This is an empirical observation that warrants further investigation. Indeed, we do not have precise data on the drainage frequency or the quantities drained during each evacuation. After the placement of the semi-permanent drain, drainage was performed every 24 to 72 h by nurses, according to the patient’s needs and discomfort. The quantities were often between 1 and 3 L. In some cases, we observed that the drainage intervals could be spaced out after a few days, with a progressive drying up of the ascites.

The average time from the diagnosis of refractory malignant ascites to death was 93.7 days [14;263], while the average time from drain placement to death was 36.5 days [4;147]. This limits our ability to fully assess the long-term benefits and complications of the procedure. Nevertheless, the number of complications in our study remains consistent with the data presented in other articles [16]. Furthermore, it may be advisable to offer the procedure earlier once refractory ascites is diagnosed. A common challenge is that when a semi-permanent peritoneal drain is indicated and the procedure is discussed with the patient, there is often a period of reflection. Additionally, there can be logistical delays related to the availability of the interventional radiology team. In collaboration with interventional radiologists, it might be beneficial to train palliative care physicians to perform semi-permanent drain placements under ultrasound guidance at the patient’s bedside similar to the existing practices for suprapubic catheters or peripherally inserted central catheters [27, 28]. This could help avoid multiple consultations and appointments with different teams, ultimately improving patient comfort and reducing the time between decision-making and procedure, especially for patients with a poor prognosis.

In our retrospective analysis, 60% of patients were able to return to home hospitalization, and none required repeat hospital visits for ascites puncture. This is a significant finding, especially for QoL. A team from the Abramson Cancer Center at the University of Pennsylvania explored this issue for patients with metastatic pancreatic adenocarcinoma, with an oncological prognosis of less than a year after diagnosis. A study of 362 patients within the University of Pennsylvania health system showed that 10% of their remaining days were spent on healthcare-related activities, with 60% of this time spent traveling to and from care or waiting for care [26]. This significantly impairs QoL. Therefore, our palliative care team strives to schedule patient consultations alongside other planned appointments to minimize repeated hospital visits and reduce the burden on patients.

## Conclusion

The management of symptoms and complications related to malignant ascites is essential for improving the QoL of affected patients. The literature on the management of malignant ascites lacks objective evaluation of the efficacy of different treatments, with low levels of evidence in studies that are often outdated.

While malignant ascites is often resistant to standard diuretic treatment, progress has been made in drainage systems. Paracentesis is the preferred therapeutic option as it is the most rapidly and consistently effective, and remains widely used. The placement of intraperitoneal catheters offers an alternative treatment for refractory malignant ascites, although there are currently no clear recommendations on this approach. When discussed, various devices are available, each with different characteristics, which guides the choice depending on the clinical situation and patient preferences. In our palliative care practice, we encounter patients with a short-term prognosis, where a non-tunneled semi-permanent catheter, with simpler implantation, may offer an advantage in terms of QoL.

This study serves as a pilot study for a prospective cohort, analyzing QoL and economic costs.

## Data Availability

No datasets were generated or analysed during the current study.
